# Comparative Study of Functionalized Cellulose Nanocrystal–Silica Aerogels for Methylene Blue Adsorption

**DOI:** 10.3390/polym17222983

**Published:** 2025-11-10

**Authors:** Nduduzo Khumalo, Samson Mohomane, Vetrimurugan Elumalai, Tshwafo Motaung

**Affiliations:** 1 Department of Chemistry, University of Zululand, Kwadlangezwa Campus, Kwadlangezwa 3886, Kwa-Zulu Natal Province, South Africa; 2 Department of Hydrology, University of Zululand, Kwadlangezwa Campus, Kwadlangezwa 3886, Kwa-Zulu Natal Province, South Africa; 3 Department of Chemistry & Chemical Technology, Sefako Makgatho Health Science University, P.O. Box 94, Medunsa 0204, South Africa

**Keywords:** cellulose nanocrystals, silica aerogel, nanocomposites, methylene blue, adsorption, wastewater treatment, carboxymethyl cellulose, kinetic studies

## Abstract

The removal of cationic dyes from industrial wastewater presents a significant environmental challenge. This research examines the effectiveness of functionalized cellulose-based silica aerogels as sustainable adsorbents for methylene blue (MB) dye. This research provides a systematic comparative study on the effectiveness of four distinct functionalization strategies, carboxylate (CCNC), double carboxylate (DCCNC), carboxymethyl (CMC), and thiol-modification, applied to cellulose-based silica aerogels as sustainable adsorbents for methylene blue (MB) dye. Cellulose nanocrystals (CNCs) were extracted from sugarcane bagasse waste and subsequently functionalized into carboxylate (CCNC), double carboxylate (DCCNC), carboxymethyl (CMC), and thiol-modified variants. The materials were later integrated into a silica matrix, resulting in the formation of porous aerogel nanocomposites. The materials underwent thorough characterization through FTIR, XRD, SEM, TGA, and BET analyses, validating successful functionalization and the development of mesoporous structures. Batch adsorption tests demonstrated that the CMC-silica aerogel exhibited superior performance, attaining a maximum adsorption capacity of 197 mg/g and complete removal efficiency under ideal circumstances (pH 10, 25 °C, 60 min). The adsorption process is accurately characterized by the Langmuir isotherm and pseudo-second-order kinetic models, signifying monolayer adsorption and chemisorption as the rate-limiting step. The thermodynamic parameters indicate that the adsorption process is exothermic and spontaneous. The CMC-silica aerogel exhibited significant reusability, maintaining over 90% efficiency after six consecutive cycles. The findings illustrate the efficacy of functionalized cellulose–silica aerogels, especially the CMC form, as effective, environmentally sustainable, and reusable adsorbents for the treatment of dye-polluted water.

## 1. Introduction

Removing cationic dyes from industrial wastewater remains a crucial yet challenging aspect of environmental remediation. The textile industry is the largest consumer, accounting for over two-thirds of the market, while the rubber, paper, and plastic industries collectively account for a significant portion of the millions of tons of cationic dyes used annually [1,2]. About 10–20% of these dyes end up in wastewater, constituting a major source of pollution. These dyes exhibit a wide range of chemical structures, and many pose a significant challenge due to their high hydrophilicity and resistance to degradation by light or heat [3]. Several methods are currently employed for dye removal, including Coagulation and flocculation [4], membrane separation [5], electrocoagulation [6], and adsorption [7]. Among these, adsorption is particularly prominent in industrial applications due to its cost-effectiveness, simplicity, and high efficiency [8,9].

Recently, there has been a lot of interest in making dye adsorbents out of cellulosic polymers because they are plentiful, renewable, and break down naturally [10,11]. Aerogels are a common type of adsorbent characterized by a highly porous, three-dimensional network structure, typically exhibiting porosity greater than 90% and high specific surface areas (often ranging from 200 to 1000 m^2^/g) [12]. This structure enables rapid swelling and exceptional dye absorption capacity.

Adsorption is an effective way of eradicating dyes [13]. A lot of cellulose-based materials have been studied as good adsorbents [14,15]. Carboxylate cellulose nanocrystals (CCNCs) are a type of cellulose nanocrystals (CNCs) that have a lot of carboxylic acid groups that are great at binding methylene blue. CNCs come from a variety of cellulosic sources, such as sugarcane bagasse, wood, straw, ramie fibre, bacterial cellulose, microcrystalline cellulose, and cotton [16]. They are stronger, more crystalline, and have a larger specific surface area than these natural celluloses [17]. As a result, different fields of research have created a variety of functional nanocomposites based on CNCs [18].

CNCs are excellent adsorbents due to their high specific surface area and abundance of active sites, making them suitable for the adsorption of dyes [19], oils [20], and heavy metal ions [9,21,22]. Functionalization of cellulose-based aerogels emerges as a promising avenue to revolutionize the purification process. Functionalization opens the door to improving the ability of these aerogels to adsorb pollutants, especially methylene blue, from polluted water by changing the properties of their surfaces [23].

Functionalization has a transformative effect that can be seen in many areas. It makes the surface area of aerogels bigger, which creates a bigger area with more active sites for methylene blue molecules to adsorb [24]. This amplification fosters heightened efficiency by accommodating a larger volume of pollutants [25]. Moreover, functionalization instigates a profound transformation in the interaction dynamics between the aerogel surface and methylene blue molecules [26]. This recalibration makes the interactions stronger and more selective, which increases the aerogels’ ability to absorb and lower the amount of methylene blue in polluted water sources. Functionalization gives the aerogels’ surfaces unique properties that are very important for improving their selectivity [27]. This selectivity enables selective targeting of pollutants, like methylene blue, with great accuracy, which improves the aerogels’ ability to clean water.

Furthermore, the optimized surface properties of functionalized aerogels are instrumental in enhancing the adsorption process [28]. The introduction of specific functional groups can significantly alter the interaction dynamics with pollutant molecules. This study is designed to systematically compare the effect of four specific functional groups—carboxylate, double carboxylate, carboxymethyl, and thiol on the adsorption of methylene blue (MB). These groups were selected to probe and contrast different adsorption mechanisms: Carboxylate groups (-COO^−^) are expected to facilitate strong electrostatic interactions with the cationic MB dye, especially at higher pH. The “carboxylate” (CCNC) and “double carboxylate” (DCCNC) variants allow us to investigate the effect of increasing the density of this primary functional group. Carboxymethyl (CMC) modification introduces a high density of carboxylate groups but on a more amorphous, swollen polymer chain, potentially enhancing accessibility and swelling capacity, which could favour both electrostatic binding and diffusion. In contrast, thiol groups (-SH) are not primarily ionic. They are expected to engage in softer Lewis acid–base interactions or potential coordination bonding with the dye, providing a distinct, non-electrostatic pathway for adsorption. These tailored interactions not only improve selectivity and capacity but can also accelerate adsorption kinetics by reducing the diffusion resistance and energy barrier for the dye molecules to bind to the surface, a phenomenon that could be quantified by changes in effective diffusion coefficients. Another important aspect is that the nature of the functional group influences regeneration and reusability; groups involved in stronger, more specific chemisorption may require different desorption conditions than those dominated by physisorption. The ability to efficiently desorb the adsorbed methylene blue enables multiple reuse cycles, making the purification process more sustainable and cost-effective [26]. Ultimately, the efficacy of functionalization depends on several factors, including the type of functional group, their density, and the synthesis conditions of the aerogel [29].

Recent studies demonstrate the efficacy of this approach. For instance, Song et al. (2019) reported that augmenting the carboxyl content on cellulose nanocrystal (CNC) surfaces remarkably enhanced their absorption and flocculation capabilities [30]. Similarly, Su et al. (2014) found that incorporating carboxylic groups improved the dispersion capacity of CNCs in water, a key factor for adsorption [31]. Lu et al. (2016) highlighted the critical role of carboxyl and hydroxyl groups on the surface of a CNC-Fe_3_O_4_ composite for adsorbing heavy metals [32]. In a direct comparison relevant to this work, Beaumont et al. (2022) showed that a thiol-functionalized cellulose nanofiber-starch-silica hydrogel adsorbed significantly more methylene blue than its non-functionalized counterpart [33].

Building on these findings, this study systematically compares the effectiveness of carboxylate (CCNC), double carboxylate (DCCNC), carboxymethyl (CMC), and thiol-functionalized cellulose nanocrystals incorporated into silica aerogel matrices for the adsorption of methylene blue (MB) dye. The primary goal is to evaluate how this specific functionalization influences the adsorption capacity and efficiency of the resulting nanocomposites.

## 2. Materials and Methods

### 2.1. Materials

Sugarcane bagasse (SCB) waste was supplied by the sugar company at Empangeni (Felixton) in KwaZulu Natal, South Africa. Methanol, Monochloroacetate, Hydrochloric Acid (32%), Sodium Chlorate (80%), Acetic acid, Ammonium persulphate (APS), TEOS (98% purity), Ammonia (25%), Citric acid, 3-mercaptopropyl) trimethoxysilane (MTMS) (95% purity), Sulfuric acid (98%), Sodium Nitrate, Sodium Hydroxide pellets, aluminum Chloride were purchased from Laboratory Supplies (South Africa) and used as received.

### 2.2. Extraction of Cellulose

**Scheme 1:**
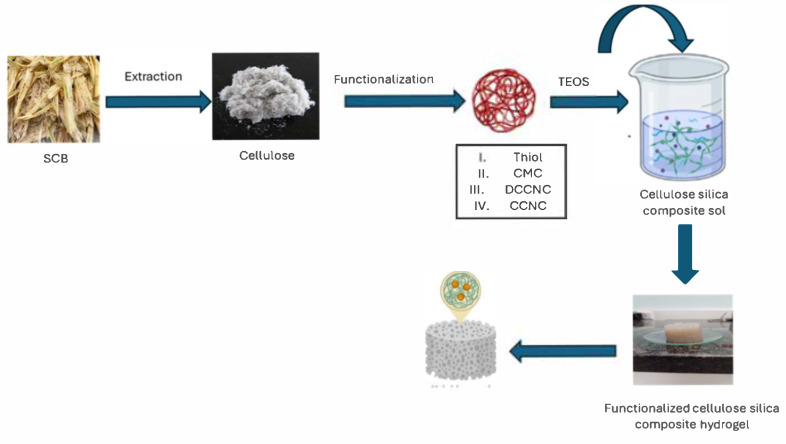
Preparation of functionalized cellulose-based aerogels.

SCB was ground up with a Fritsch cutting mill pulveriser 15. After that, it was boiled in distilled water for two hours and then dried in an oven at 55 °C overnight (Scheme 1). The dried SCB was treated with 4 wt% NaOH at 80 °C for an hour, then rinsed with distilled water. This process was done twice. The SCB that had been treated with alkali was then dried in an oven at 55 °C.

After being treated with alkali, this SCB was bleached for an hour at 80 °C using a buffer solution made of 54 g of NaOH and 150 mL of CH_3_COOH mixed with 2 L of a 1.7 wt% NaClO_2_ solution. This step was done twice more. Finally, the bleached cellulose was filtered, washed with distilled water until the pH was neutral, and then dried overnight at 55 °C.

### 2.3. Preparation of Carboxylate Cellulose Nanocrystals

A mass of 18 g of dry cellulose was added to a 600 mL solution of ammonium persulfate (APS) at a concentration of 2 M. Ultrasound was applied to the mixture for four hours at a temperature of 70 °C (Power 005) utilizing high frequency and continuous agitation. Subsequent to achieving homogeneity, the mixture was centrifuged with distilled water at 6000 rpm for four cycles. Subsequently, the suspension was dialyzed against distilled water until the pH of the CCNC solution approached neutrality.

#### Preparation of CCNC-Silica Aerogels

A mixture consisting of 2.8 mL of 1.7 wt% CCNC dispersion (equivalent to 0.12 g dry cellulose), 9.2 mL of water, 2 mL of TEOS (98% purity, 1.9 g, 9.1 mmol), and 0.16 mL of aqueous HCl (0.29 M, 46 µmol HCl) was stirred overnight to facilitate the hydrolysis of TEOS, leading to the formation of the CCNC-silica sol. Subsequently, 0.85 mL of 0.1 mol/L NH_3_ (85 µmol) was added and mixed to initiate condensation. The solution was subsequently transferred into syringes to create hydrogels with cylindrical forms, which can be modified as needed using various molds. Upon achieving complete gelation, the silica nanocomposites were aged in water at 50 °C for at least 10 h to improve the stiffness of the silica gel network. The hydrogel was subjected to vacuum drying at 30 °C to produce aerogels, which were then maintained under vacuum conditions until use.

### 2.4. Preparation of Double Carboxylate Cellulose Nanocrystals

The CCNC obtained was dispersed in a 1 mol L^−1^ citric acid solution (200 mL) and subjected to ultrasonication, followed by stirring for an additional 2 h at 60 °C. The product was subsequently washed via centrifugation using deionized water. The material was then redispersed in a moderate volume of deionized water and subjected to dialysis against deionized water for about one week to obtain a nearly neutral suspension of double carboxylate cellulose nanocrystals (DCCNC).

#### Preparation of DCCNC-Silica Aerogel

A mixture consisting of 3.8 mL of 1.7 wt% DCCNC dispersion (equivalent to 0.12 g dry cellulose), 8.2 mL of water, 2 mL of TEOS (98% purity, 1.9 g, 9.1 mmol), and 0.16 mL of aqueous HCl (0.29 M, 46 µmol HCl) was stirred overnight to facilitate the hydrolysis of TEOS, yielding the DCCNC-silica sol. A procedure analogous to Preparation of CCNC-Silica Aerogels Section was subsequently employed to synthesize DCCNC-silica aerogel.

A mixture consisting of 3.8 mL of 1.7 wt% DCCNC dispersion (equivalent to 0.12 g dry cellulose), 8.2 mL of water, 2 mL of TEOS (98% purity, 1.9 g, 9.1 mmol), and 0.16 mL of aqueous HCl (0.29 M, 46 µmol HCl) was stirred overnight to facilitate the hydrolysis of TEOS, yielding the DCCNC-silica sol. A procedure analogous to Preparation of CCNC-Silica Aerogels Section was subsequently employed to synthesize DCCNC-silica aerogel.

### 2.5. Preparation of Carboxymethyl Cellulose

A mass of 10 g of cellulose was dispersed in a 30% NaOH solution, followed by the addition of 67 mL of ethanol to the mixture. The mixture was stirred for 90 min at 35 °C, after which the temperature increased to 70 °C with the addition of chloroacetic acid for a duration of 2 h. The mixture was subsequently cooled to room temperature. Following cooling, the blend underwent neutralization with acetic acid, filtration, and several washes with 80% ethanol. The CMC product was dried overnight at 55 °C in an oven.

#### Preparation of CMC-Silica Aerogel

A mixture consisting of 0.12 g of CMC, 9.2 mL of water, 2 mL (1.9 g, 9.1 mmol) of TEOS (98% purity), and 0.16 mL (0.29 M, 46 µmol) of aqueous HCl was stirred overnight to facilitate the hydrolysis of TEOS, yielding a CMC-silica sol. A procedure analogous to Preparation of CCNC-Silica Aerogels Section was subsequently employed to synthesize CMC-silica aerogel.

### 2.6. Preparation of Thiol-Functionalized CCNC-Silica Aerogel

The aerogels were produced using the previously established method for creating CCNC-silica aerogels, employing the same proportions of materials. After acidic hydrolysis, (3-mercaptopropyl) trimethoxysilane (MTMS) (95% purity, 0.13 mL, 0.14 g, 0.7 mmol) was added to the prepared CCNC-silica sol before the introduction of ammonia. Aluminium foil was employed to protect the sample from light following the addition of mercapto silane. Subsequently, 0.85 mL of 0.1 mol/L NH_3_ (85 µmol) was added, and the sample was placed into a mould. Following complete gelation, the silica nanocomposites were aged in water at 50 °C for at least 10 h to enhance the network structure. The hydrogels were subjected to vacuum drying at 30 °C for 48 h to produce aerogels, which were then maintained under vacuum conditions until use.

### 2.7. Characterization Methods

#### 2.7.1. Fourier Transform Infra-Red Spectroscopy

Samples were analyzed with a Perkin Elmer attenuated total reflection FTIR spectrometer (Perkin Elmer UATR Two) in diffuse reflectance mode to conduct Fourier transform infrared spectroscopy (FTIR) on the samples. The spectral range employed for sample analysis was 4000 to 500 cm^−1^.

#### 2.7.2. X-Ray Diffraction

The X-ray diffraction (XRD) analysis of the samples was conducted using a Bruker AXS Advance D8 diffractometer located in Karlsruhe, Germany. The instrument utilized a monochromatic Cu Kα X-ray source (λ = 1.5406 Å), operating at 40 kV and 40 mA under room temperature conditions. The Segal empirical method and the deconvolution method were employed to ascertain the crystallinity index (CI). The Segal empirical method calculates the crystallinity index (CI) using the heights of the I_002_ and I_min_ peaks, which are situated between the 002 and 001 peaks. The confidence interval calculation was performed using the method outlined below [34]:

(1) $$CI\;\%=\frac{I002-Iam\times 100}{I002}$$

In this case, I002 stands for the highest diffraction intensity at the peak, and Iam stands for the intensity of diffraction from the amorphous material [34].

The deconvolution method calculates the CI by looking at the ratio of the total area to the combined area of all the crystalline peaks.

(2) $$CI\;\%=\frac{\sum Acryst\times 100}{\sum Acryst+\sum Aamorp}$$

In this case, Acryst stands for the area assigned to the crystalline domain, and Aamorp stands for the area assigned to the amorphous domain.

#### 2.7.3. Scanning Electron Microscopy

The FEI Quanta 200 electron microscope, operated at an acceleration voltage of 20 kV, was utilized for SEM measurements of the samples. The samples were coated with carbon using Edward’s E306A coating system prior to analysis.

#### 2.7.4. Thermogravimetric Analysis

A thermogravimetric analysis (TGA) was conducted on the samples using a Perkin Elmer Pyris 6 analyzer. The samples, weighing between 10 and 15 mg, were subjected to heating from 30 to 700 °C at a rate of 5 °C per minute. The process occurred in a nitrogen environment at a flow rate of 20 mL/min.

### 2.8. Adsorption Experiments

Batch adsorption experiments were performed to evaluate the adsorption process. A precise mass of aerogels, ranging from 0.1 to 0.4 g, was incorporated into 50 mL of methylene blue dye solution, with concentrations varying from 50 to 200 mg/L. The pH was modified from 2 to 12 through the addition of 0.1 M solutions of HCl and NaOH. The tests were conducted under constant stirring conditions at a temperature of 301 K. The residual concentration of MB dye was quantified at 665 nm using a Pharo 300 Spectroquant UV–vis spectrophotometer at specified intervals of 30, 60, 90, 120, 150, 180, 210, and 240 min. The equations employed to determine the removal percentage (R%) and the adsorption capacity (qt) are presented below:

(3) $$R\;(\%)=\frac{C0-Ct\times 100}{C0}$$

(4) $$qt\;(mg/g)=\frac{(C0-Ct)\times V}{M}$$

C0 and Ct (mg/L) represent the MB concentrations at the initial (0) time and the final time, respectively. V represents the volume of the MB solution in litres (L), while M denotes the net weight of the dried cellulose-based silica aerogels in grams (g). To renew and reuse the MB-loaded aerogels, they were collected and cleaned using a regeneration solution, which is a diluted acid that desorbs the MB dye. The aerogels were then dried at 50 °C in a vacuum oven and used again in the following cycles to evaluate their effectiveness after multiple uses.

## 3. Results and Discussion

### 3.1. FTIR Spectroscopy Analysis

**Figure 1:**
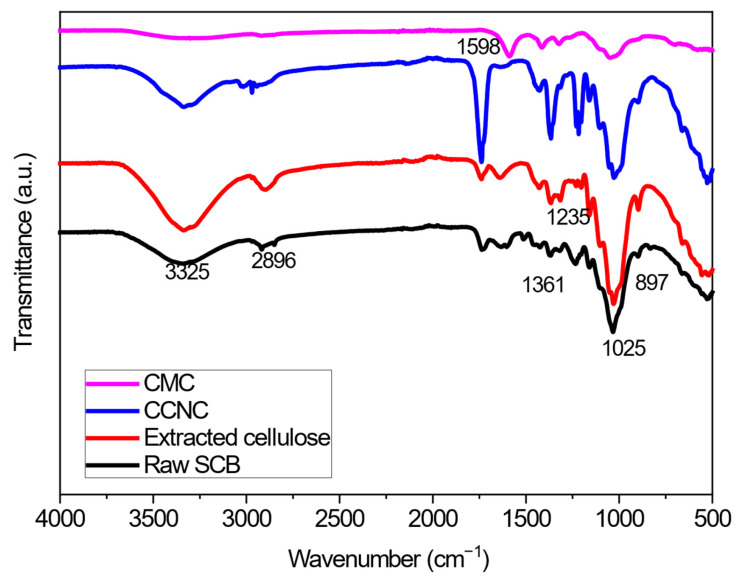
FTIR spectra of raw SCB, extracted cellulose, and various functionalized cellulose.

The FTIR analysis aimed to identify the functional groups in carboxylate cellulose, carboxymethyl cellulose, and the aerogels that go with them. Figure 1 shows FTIR spectra for raw sugarcane bagasse (SCB), extracted cellulose, CCNC, and CMC. In raw SCB, there were clear peaks at 3325 cm^−1^ (–OH stretching), 2896 cm^−1^ (C–H vibrations), 1361 cm^−1^ (–CH_2_ bending), 1025 cm^−1^ (C–O stretching), and 897 cm^−1^ (–CH_2_ bending) that are associated to cellulose I [35,36,37,38].

There was an additional peak at about 1312 cm^−1^ in extracted cellulose, and CCNC, but not in raw SCB. This peak is likely caused by C–H wagging, which happens when hydrogen bonds are broken [39]. The extracted cellulose had identical peak patterns as the raw SCB, but there was an extra peak around 1312 cm^−1^. However, no peaks were observed at 1508 and 1239 cm^−1^, but it did have higher peak intensity at 897 cm^−1^ between glucose units in cellulose, which means that the amorphous areas in cellulosic materials were removed [36,39].

**Figure 2:**
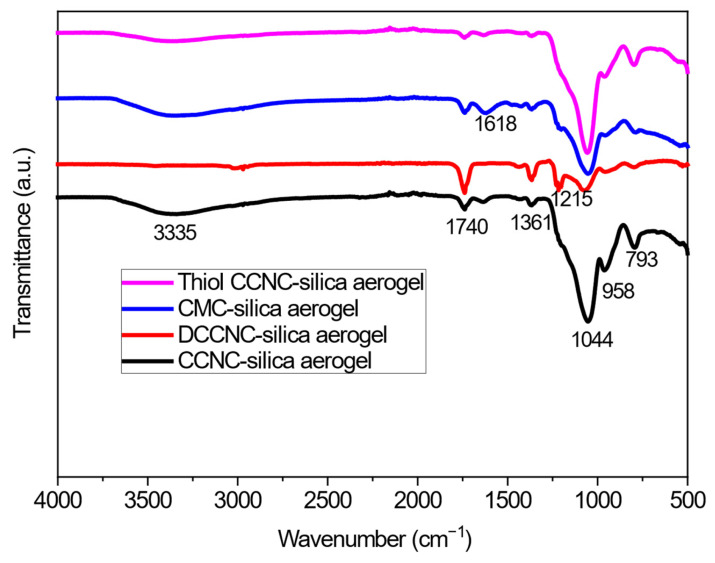
FTIR spectra of functionalized cellulose-based silica aerogels.

Successful functionalization is evidenced by the appearance and intensification of new peaks. The introduction of carboxyl groups via APS oxidation (CCNC) is confirmed by the pronounced increase in the intensity of the carbonyl (C=O) stretching band at approximately 1740 cm^−1^ [40]. This peak is further intensified in the DCCNC-silica aerogel (Figure 2) spectrum, confirming the success of the double carboxylation treatment with citric acid. In contrast, the CMC spectrum is dominated by a strong, broad band at 1598 cm^−1^, assigned to the antisymmetric stretching of the carboxylate ion (-COO^−^), alongside the characteristic C-O stretching at ~1025 cm^−1^, which is consistent with the carboxymethylation reaction [41].

Figure 2 shows the FTIR spectra for CCNC-silica, DCCNC-silica, CMC-silica, and thiol-functionalized CCNC-silica aerogels. The broad O-H stretching band at ~3335 cm^−1^ was less intense in the CCNC-, DCCNC-, and thiol-CCNC-silica aerogels compared to their pure cellulose counterparts. This reduction is attributed to the formation of the silica network, which can form hydrogen bonds with cellulose hydroxyls, potentially altering the band’s characteristics, and to the dilution effect of the silica matrix. In contrast, the CMC-silica aerogel exhibited a more complex profile in this region. The extensive carboxymethylation of the cellulose backbone significantly alters its hydrogen-bonding network, which, combined with its interaction with silica, results in a distinct O-H band shape and intensity compared to the other aerogels. The FTIR analysis of all the aerogels showed a clear peak at 793 cm^−1^, which was caused by the stretching and bending vibration of the silanol group (Si-OH) [41]. Also, peaks at 1361 cm^−1^, 1215 cm^−1^, and 1044 cm^−1^ were linked to the stretching of C–O alkoxy, C–O–C asymmetric stretch vibration, and C–O epoxy, respectively. This showed that cellulose-based silica aerogels had been made successfully [41].

### 3.2. X-Ray Diffraction Analysis

**Figure 3:**
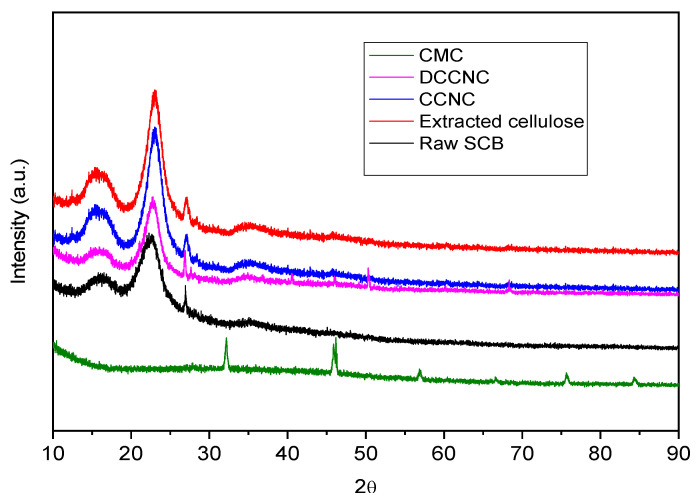
XRD spectra of raw SCB, extracted cellulose, and various functionalized cellulose.

Figure 3 shows XRD spectra with raw SCB, extracted cellulose, CCNC, DCCNC, CMC, and thiol-functionalized CCNC. The X-ray diffraction (XRD) results show that the crystalline structure of cellulose changes a lot at different stages of processing sugarcane bagasse. The presence of lignin, hemicellulose, and other non-cellulosic materials in raw sugarcane bagasse makes it look like a broad amorphous hump. There are also weak crystalline peaks of cellulose I around 2θ = 15° and 22.5° [42]. After extraction, removing the amorphous parts makes the crystals more crystalline, which makes the peaks at 2θ = 15°, 16.4°, and 22.5° sharper and more intense. These peaks are for the (101), (10ī), and (002) planes of cellulose I (Table 1) [43,44]. Adding carboxyl groups to cellulose to make carboxylate cellulose causes some of the crystallinity to break down, as shown by the peaks becoming wider and less intense. This means that some of the cellulose has become amorphous [45,46]. This disruption gets worse in double carboxylate cellulose, where more carboxyl groups make the peaks even wider and weaker, showing a higher level of amorphization [47,48]. CMC, when modified more strongly, has a mostly amorphous structure with very weak or no clear crystalline peaks [49].

The alterations in the XRD patterns are assessed by the Crystallinity Index (CI), which increases from raw bagasse to extracted cellulose and decreases with each successive modification. Although peak placements stay largely stable, the intensities and widths of these peaks fluctuate, signifying differing levels of crystallinity and structural disturbance at each processing stage. The results underscore the structural alterations essential for comprehending the material qualities and prospective applications of modified cellulose obtained from sugarcane bagasse. To execute that procedure.

**Figure 4:**
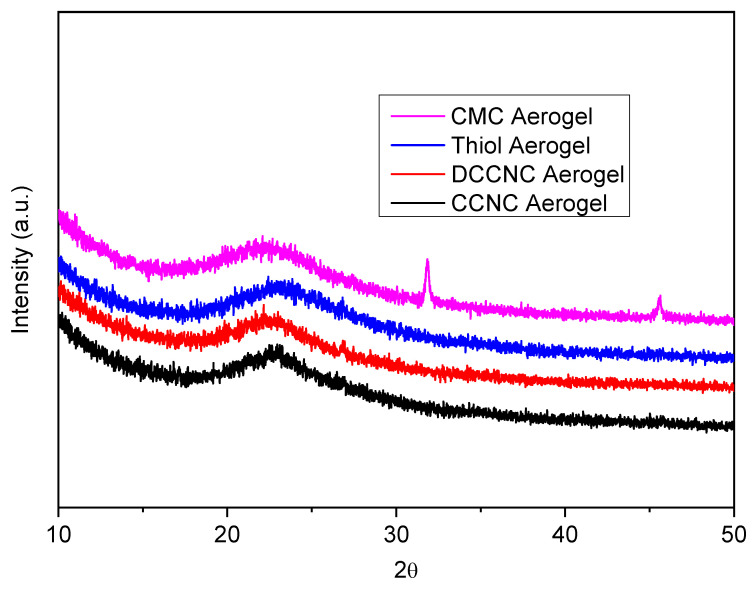
XRD spectra of functionalized cellulose-based silica aerogels.

Figure 4 shows the XRD results for different types of functionalized cellulose nanocrystal (CNC) silica aerogels. Carboxylate CNC (CCNC) silica aerogel, double carboxylate CNC (DCCNC) silica aerogel, thiol-functionalized CCNC silica aerogel, and carboxymethyl cellulose (CMC) silica aerogel show how adding chemicals and silica changes the way their crystals look. The XRD pattern of the CCNC silica aerogel is dominated by a very broad hump centered around 2θ = 22°, which is characteristic of amorphous silica [38,45,46,50]. The characteristic crystalline peaks of cellulose I, which are visible in the pure CCNC material (Figure 4), are significantly suppressed and broadened in the aerogel composite. This indicates that the crystalline structure of the cellulose nanocrystals is largely masked by the overwhelming amorphous silica matrix. The persistence of a low-intensity, broad feature in the region of 22.5° suggests that some residual cellulose I crystallinity may be present, but the long-range order is greatly disrupted upon incorporation into the silica network. The peaks in DCCNC silica aerogels get wider and less intense, which means that the crystalline structure is more broken up [47,48]. Thiol-functionalized CCNC silica aerogels cause even more disruption, with peaks that are much wider and lower [46]. Most of the CMC silica aerogels are amorphous, with very weak cellulose peaks that show a lot of disruption. However, they still have broad peaks around 2θ = 20–30° from amorphous silica [49]. As the chemical modification and structural disruption increase, the Crystallinity Index (CI) goes down from CCNC to DCCNC to thiol-functionalized CCNC to CMC silica aerogels (Table 2). The peaks stay in the same places, which shows that the basic cellulose structure is still there. However, the heights and widths of these peaks change, showing that the crystallinity is different. These results are in line with what other studies have found about how chemical changes affect the crystalline structure of cellulose and how it interacts with silica [45,46,47,48,49].

### 3.3. SEM Analysis

**Figure 5:**
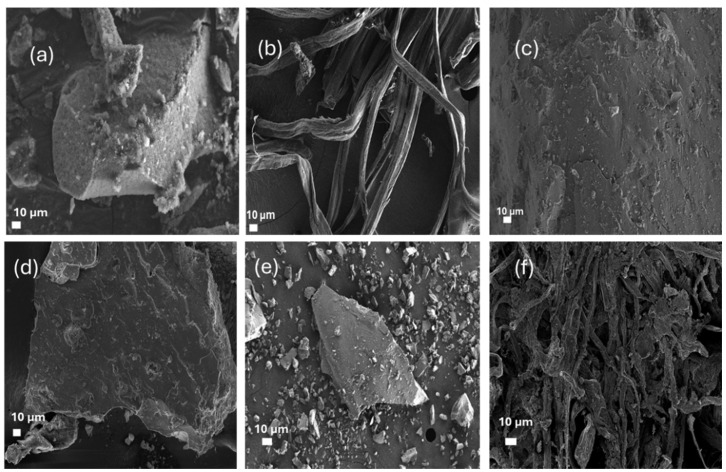
SEM images of (**a**) raw SCB, (**b**) extracted cellulose, (**c**) CCNC silica aerogel, (**d**) DCCNC silica aerogel, (**e**) Thiol silica aerogel, and (**f**) CMC silica aerogel.

Figure 5 presents scanning electron microscopy (SEM) images that provide critical insights into the morphological variations of cellulose during various stages of extraction and functionalization, along with the fabrication process of silica aerogel composites. The surface of raw sugarcane bagasse (SCB) exhibits a coarse and irregular roughness comprising both fibrous and non-fibrous components. This is due to the presence of lignin, hemicellulose, and other amorphous components [36,42]. The extracted cellulose (Figure 5b) exhibits a purer, more fibrillar structure with reduced surface detritus following alkali and bleaching treatments. This indicates that the majority of non-cellulosic materials have been eliminated [48]. The SEM images illustrate the transformation of shape from raw biomass to the final aerogel composites. The incorporation of cellulose derivatives into silica matrices results in structures distinct from fundamental fibrillar cellulose. The CCNC-silica (Figure 5c), DCCNC-silica (Figure 5d), and thiol-CCNC-silica (Figure 5e) aerogels exhibit diverse morphologies, characterized by regions of aggregated particles and the development of a macroporous network [12,23]. The thiol-functionalized aerogel (Figure 5e) demonstrates enhanced density, presumably due to the inclusion of MTMS during its production. The CMC-silica aerogel (Figure 5f) demonstrates a more homogeneous and cohesive gel structure, distinguished by finer, sponge-like porosity [23]. The unique shape and amorphous traits evident in XRD are likely attributable to the behaviour of the dissolved CMC polymer during gelation inside the silica matrix, in contrast to the dispersion patterns of CNC variants. The SEM images at this magnification indicate that the aerogels exhibit distinct differences from one another [31,51]. The BET analysis (Table 3) indicates significant mesoporosity in all cases, which is crucial for their adsorption capacity. The distinctive, finer porous structure of CMC-silica aerogel is particularly effective for adsorbing methylene blue, since it likely offers an optimal combination of surface area and pore accessibility [51].

### 3.4. TGA Analysis

**Figure 6:**
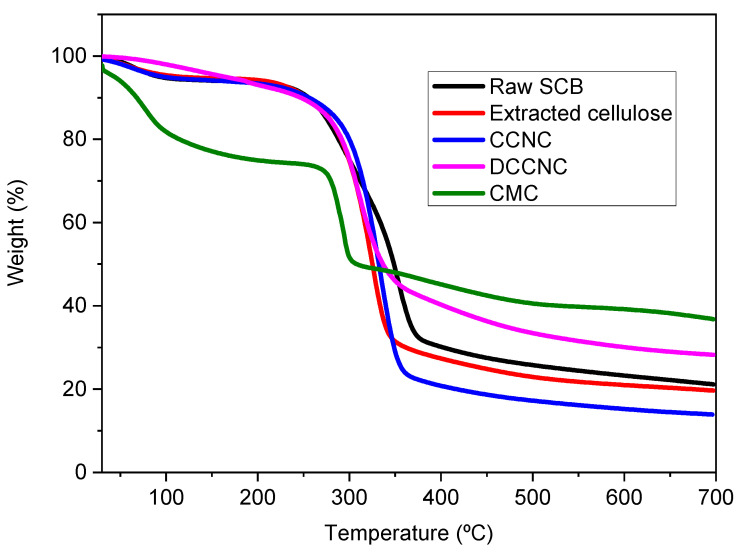
TGA spectra of raw SCB, extracted cellulose, and various functionalized cellulose.

**Figure 7:**
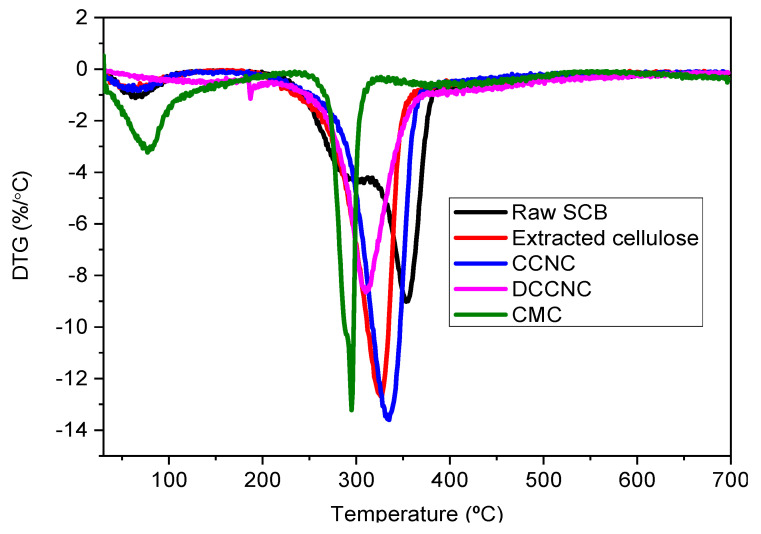
DTG spectra of raw SCB, extracted cellulose, and various functionalized cellulose.

Thermogravimetric analysis was used to study changes in the thermal properties of raw SCB, extracted cellulose, CCNC, DCCNC, CMC, and thiol-functionalized CCNC. Figure 6 and Figure 7 show the TGA and DTG curves, respectively. Three distinct degradation steps were identified in raw sugarcane bagasse (SCB). The first step, which happened at about 100 °C, was related to the removal of moisture. The second step, which took place at temperatures between 270 and 290 °C, involved getting rid of hemicellulose, waxes, and pectin. The third step, which took place at temperatures between 320 and 354 °C, was caused by the breakdown of lignin [16,52,53,54].

On the other hand, all the other materials that were studied showed two steps of degradation. The first one happened at about 100 °C, which meant that the moisture was evaporating. For extracted cellulose, the second step of degradation happened between 300 and 326 °C. For CCNC, it showed up between 310 and 334 °C, and for DCCNC, it showed up between 280 and 309 °C. This means that non-cellulosic materials were removed and that the thermal stability of the material was slowly decreasing compared to raw SCB [36]. These results point to less thermal stability during oxidation. For CMC, its TGA curve showed two steps of degradation at about 100 °C and 250–290 °C. This means that moisture was removed, and possibly carbon dioxide was released from the polymer chain [51].

**Figure 8:**
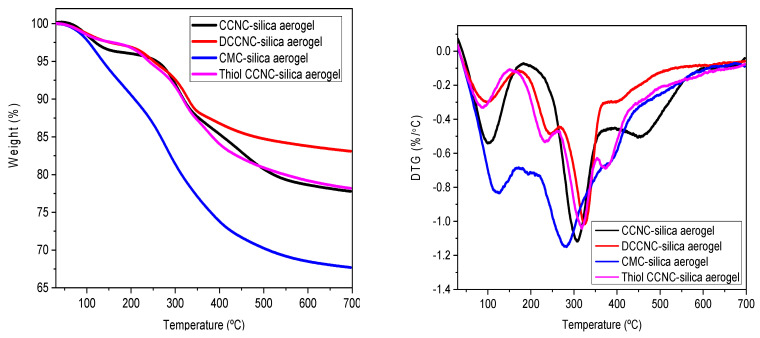
TGA and DTG spectra of functionalized cellulose-based silica aerogels.

TGA was also used to study how adding silica to functionalized cellulose affects the thermal stability of cellulose-based silica aerogels. Figure 8 shows the TGA and DTG curves, respectively. The Thermogravimetric Analysis (TGA) of the CCNC-silica aerogel showed that the weight loss started at 100 °C, mostly because water evaporated. Then, around 260–310 °C, there was a second step of degradation that was caused by dehydration and the release of volatile compounds like carbon dioxide and acetic acid [41]. There was a third step of degradation between 400 and 453 °C, and the ash content was about 78% at 700 °C. The silica in the aerogels is the reason for the high ash content as compared to the functionalized cellulose ones. The DTG curves for the DCCNC and Thiol CCNC-silica aerogels reveal a more complex multi-peak profile, which provides insight into the different components and bonds within the composite. The peak around 230–250 °C, which is more pronounced in these aerogels, can be attributed to the thermal breakdown of the more labile, densely grafted carboxyl groups from citric acid treatment (in DCCNC) and the organosilane framework from MTMS (in Thiol-CCNC). Thiol groups (-SH) and the propyl chains of MTMS have different thermal stability compared to the cellulose backbone, leading to this distinct lower-temperature degradation event. The subsequent major peak (e.g., ~260–310 °C) corresponds to the main chain scission of the cellulose component, while the high-temperature event (>400 °C) is associated with the silica network, as seen in the CCNC-silica sample. The CMC-silica aerogel exhibited the lowest thermal stability among the composites, with three clear degradation steps and a final ash content of about 67% at 700 °C. This is consistent with the inherently lower thermal stability of the heavily modified, amorphous CMC polymer.

### 3.5. BET Analysis

**Figure 9:**
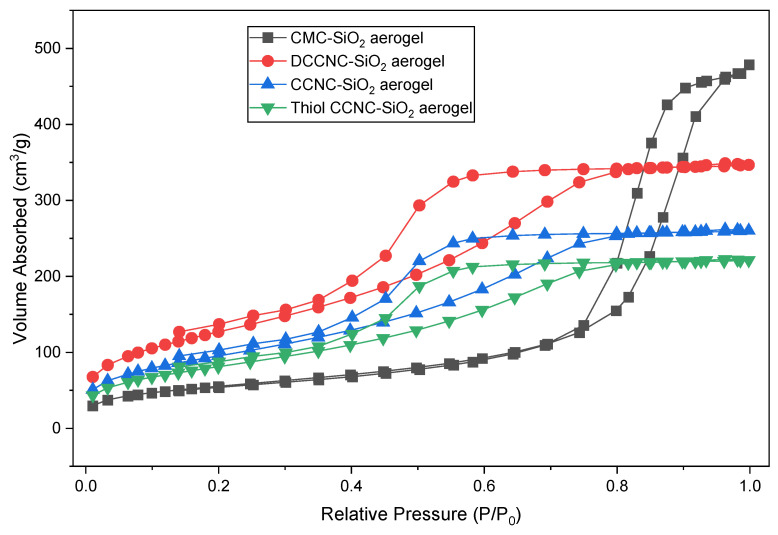
Nitrogen adsorption and desorption isotherms plots of functionalized cellulose-based silica aerogels.

The nitrogen adsorption–desorption isotherms for CMC-SiO_2_, DCCNC-SiO_2_, CCNC-SiO_2_, and thiol-functionalized CCNC-SiO_2_ aerogels (Figure 9) show type IV isotherms with H3-type hysteresis loops. This is typical of mesoporous materials found in silica-based aerogels [12,23]. DCCNC-SiO_2_ had the highest specific surface area (446.86 m^2^/g) and the smallest average pore diameter (4.59 nm) of all the samples. This means that it has a lot of uniform mesopores that are close together. This could mean that there are more places for dye molecules to stick to the surface, but it could also mean that bigger dye molecules move more slowly. The surface area of CMC-SiO_2_ was smaller (195.06 m^2^/g), but the pore diameter was larger (14.33 nm), which made it easier for methylene blue to spread through the porous network more quickly. This matches its high adsorption capacity in Section 3.6.

The CCNC-SiO_2_ aerogel probably has average properties. Its surface area is about 312.00 m^2^/g, its pore volume is 0.60 cm^3^/g, and its pore diameter is 7.70 nm. The fact that CCNC-SiO_2_ has a large surface area and pores that are easy to access makes it a good place for both dye adsorption and diffusion. The surface area (about 280.12 m^2^/g) and pore volume (about 0.55 cm^3^/g) of thiol-functionalized CCNC-SiO_2_ were both a little smaller than those of CCNC-SiO_2_. This was likely because the thiol groups that were added during functionalization blocked some of the pores. The structure was still mesoporous, though, with a pore diameter of 7.90 nm.

The BET results are the same as what was seen in the SEM (Section 3.3). This showed that the porous networks were linked in different ways depending on how they were functionalized. The results from Section 3.6 [24,38] show that these aerogels can hold more methylene blue because they have a larger specific surface area and the right size distribution of mesopores.

#### The Correlation Among Surface Area, Functionalization, and Adsorption Efficacy

The BET study reveals that the specific surface area is not the sole determinant of adsorption capacity. The DCCNC-SiO_2_ aerogel possesses the highest specific surface area (446.86 m^2^/g), but the CMC-SiO_2_ aerogel exhibits a significantly lower surface area (195.06 m^2^/g) and a markedly greater adsorption capacity for methylene blue (197 mg/g compared to 37 mg/g). The seeming paradox can be clarified by the combined effect of three key factors: functional group density, accessibility, and the physicochemical properties of the adsorbent.

Enhanced functional group density and accessibility: The carboxymethylation process introduces many carboxylate groups (-COO^−^) directly onto the cellulose backbone. Functional groups predominantly reside on the surface of crystalline nanoparticles within CNC-based aerogels. Conversely, the CMC polymer is extensively functionalized inside its amorphous, swelling matrix. This provides the cationic MB molecules with significantly more active sites for electrostatic binding per unit mass of adsorbent. This is superior to other adsorbents with greater surface areas.

Enhanced Swelling and Pore Permeability: The CMC polymer exhibits significant hydrophilicity and undergoes substantial swelling in aqueous environments. The swelling process can enlarge the mesoporous network (with a pore diameter of 14.33 nm), facilitating the accessibility of dye molecules to the numerous internal binding sites. CCNC and DCCNC possess more stiff, nanocrystalline structures that may feature smaller, less accessible pores (4.59–7.70 nm). This complicates the diffusion and attachment of bigger dye molecules, such as MB, to the interior of the structures, despite their greater nominal surface area.

The mean pore diameter of CMC-SiO_2_ (14.33 nm) facilitates the rapid diffusion and accommodation of methylene blue molecules (estimated dimensions ~1.4 × 0.6 × 0.3 nm) more effectively than the smaller pores of DCCNC-SiO_2_ (4.59 nm). This diminishes the resistance to mass transfer, facilitating a more rapid and complete approach to equilibrium for the adsorbate, as demonstrated by the kinetic studies.

### 3.6. Factors Affecting Methylene Blue Dye Adsorption

For the purpose of optimizing the adsorption parameters, a material was chosen at random, and then the optimized conditions were applied to the remaining materials that were generated. There is a presentation of the findings in Table 4.

#### 3.6.1. Effect of Contact Time

**Figure 10:**
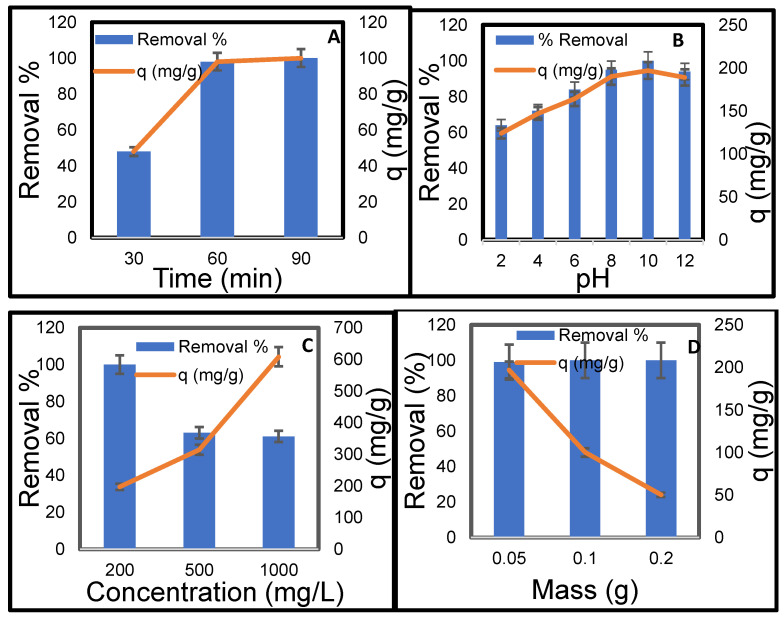
Effect of (**A**) contact time, (**B**) pH, (**C**) initial concentration of MB, and (**D**) Temperature on the adsorption of MB on CMC-silica aerogel composite [Conc. 200 mg/L, Volume 50 mL, Mass 0.1 g, Temperature 25 °C].

Figure 10A illustrates the effect of contact time on the removal efficiency and adsorption capacity of methylene blue (MB) dye on CMC-silica aerogel. The data demonstrate that extending contact time to 60 min significantly enhances both the removal percentage and adsorption capacity of MB dye. During the first 30 min, the removal percentage rapidly rises to 48%, with an adsorption capacity of 48 mg/g. A similar increase is observed from 30 to 60 min, achieving 98% removal and an adsorption capacity of 98 mg/g. This development is attributed to the numerous initially vacant sites on the CMC-silica aerogel. At the conclusion of the 60 min interval, only 2% of MB remains in the solution, which is eliminated within 90 min, indicating effective dye removal during this period.

#### 3.6.2. Effect of pH

The pH of the solution is crucial as it influences both the surface charge of the adsorbent and the composition of the dye molecule. Figure 10B illustrates the influence of pH on the adsorption capacity of MB onto the CMC-silica aerogel. As the pH increased from 2 to 10, the adsorption capacity significantly rose from 124 mg/g to 197 mg/g. Under highly acidic conditions (pH 2), the carboxylate groups (-COO^−^) on the CMC become protonated to their neutral -COOH form, resulting in diminished electrostatic interactions. Furthermore, MB can be protonated to form a neutral species (MBH^2+^ → MBH_3_^2+^ or neutral leuco form) at low pH, which diminishes the interaction between cations and anions. The high adsorption (64% removal) seen at pH 2 suggests that non-electrostatic interactions, such as hydrogen bonding between the MB molecule and the hydroxyl/silanol groups of the aerogel, along with π-π interactions, play a crucial role in the adsorption process [55]. The carboxylate groups dissociate their protons when the pH increases, resulting in a significantly negative charge on the aerogel surface. Simultaneously, MB predominantly exists in its cationic form (MB^+^). This generates a robust electrostatic attraction, which serves as the primary mechanism for optimal adsorption at pH 10 [56]. The slight decrease in adsorption at pH > 10 may be attributed to an increased presence of OH^−^ ions competing with dye anions for adsorption sites, or it may result from the potential instability of the aerogel in highly alkaline conditions.

#### 3.6.3. Effect of Initial Methylene Blue Concentration

Figure 10C shows how the initial concentration affects the substance’s removal percentage and adsorption capacity (q). The removal efficiency is highest at an initial concentration of 200 mg/L, reaching 100%. This means that there are enough active sites to capture all the available molecules. At this concentration, the adsorption capacity is 197 mg/g. When the starting concentration goes up to 500 mg/L, the percentage of removal goes down to 63%. This suggests that the number of available adsorption sites is a limiting factor [57]. The adsorption capacity increases to 314 mg/g, indicating that a greater initial concentration results in enhanced adsorption per unit mass, despite a decrease in process efficiency. The removal rate drops to 61% when the initial concentration is at its greatest, which is 1000 mg/L. This goes along with the assumption that site saturation makes it harder to get rid of more. The adsorption capacity is 608 mg/g, which is the greatest of the three concentrations, even if the removal efficiency is lower. This means that the adsorbent keeps absorbing more chemicals at higher concentrations, but it does so less and less well with time. As the starting concentration increases, the rate of removal goes down, while the adsorption capacity goes up. This happens because the adsorption sites get full, which lets the adsorbent hold on to more material at higher concentrations [58].

#### 3.6.4. Effect of Adsorbent Dose

Figure 10D shows how different masses of dry CMC-silica aerogel (from 0.05 to 0.2 g) affect the adsorption of MB dye while keeping everything else the same. The removal rate remained at 100% as the amount of adsorbent increased from 0.05 g to 0.2 g. This happened while the adsorption capacity went down from 197 mg/g to 50 mg/g. This change in behaviour is due to the fact that there are more surface sites available for MB adsorption [38,59]. On the other hand, higher doses of the adsorbent cause more particles to stick together, which lowers the adsorbent’s ability to hold onto particles [37].

#### 3.6.5. Effect of Temperature

**Figure 11:**
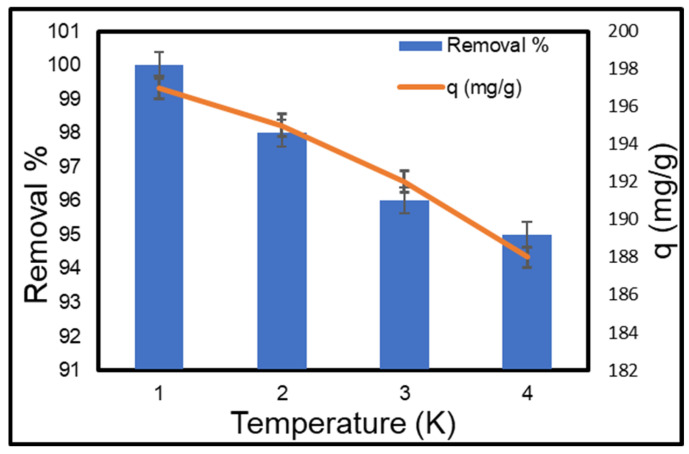
Effect of temperature on the adsorption of MB on CMC-silica aerogel composite.

The influence of temperature was examined at 298, 308, 318, and 328 K, while all other variables were held constant. Figure 11 shows that the adsorption capacity dropped steadily from 197 mg/g to 124 mg/g as the temperature rose from 298 K to 328 K. This indicates that the adsorption process of MB dye onto the CMC-silica composite aerogel is exothermic. These results are in line with what Li et al. [60] found in a study that came out before this one.

### 3.7. Adsorption Isotherm

The adsorption isotherm models were used to investigate how well cellulose-based silica aerogels adsorb and how the adsorbent and adsorbate interact with each other [61]. There are a lot of different isothermal models, but the Langmuir and Freundlich models are the ones that are used the most [62,63]. The Langmuir model says that single-layer adsorption makes a uniform surface. Its equation is shown below:

(5) $$\frac{C_{e}}{q_{e}}=\frac{C_{e}}{q_{m}}+\frac{1}{q_{m}k_{L}}$$

In this context, *C*_*e*_ (mg/L) indicates the equilibrium concentration of MB, *q*_*e*_ (mg/g) signifies the quantity of MB dye adsorbed onto the surface of the functionalized cellulose-based silica aerogels at equilibrium, *q*_*m*_ (mg/g) represents the maximum adsorption capacity of the MB dye on the adsorbent surface, and *K*_*L*_ (L/mg) denotes the Langmuir constant. The parameters *q*_*m*_ and *K*_*L*_ were derived from the slope and intercept of the *C*_*e*_/*q*_*e*_ versus *C*_*e*_ graph. The equilibrium parameter *R*_*L*_ was calculated using the following equation to evaluate the favourability of the adsorption process [64].

(6) $$R_{L}=\frac{1}{\left(1+K_{L}C_{0}\right)}$$

where *C*_0_ represents the initial concentration of MB. The Freundlich model, indicating a heterogeneous adsorbent surface, is expressed by the following equation:

(7) $$\ln qe=\ln K_{F}+\frac{1}{n}\ln C_{e}$$

**Figure 12:**
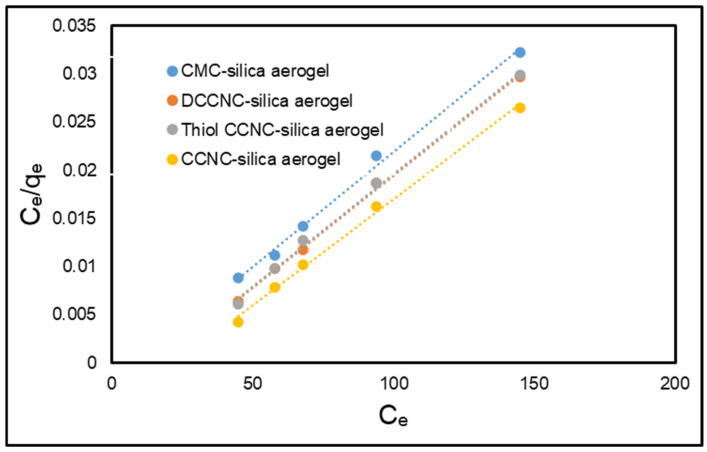
Langmuir isotherm model for the adsorption of MB onto functionalized cellulose-based silica aerogels.

**Figure 13:**
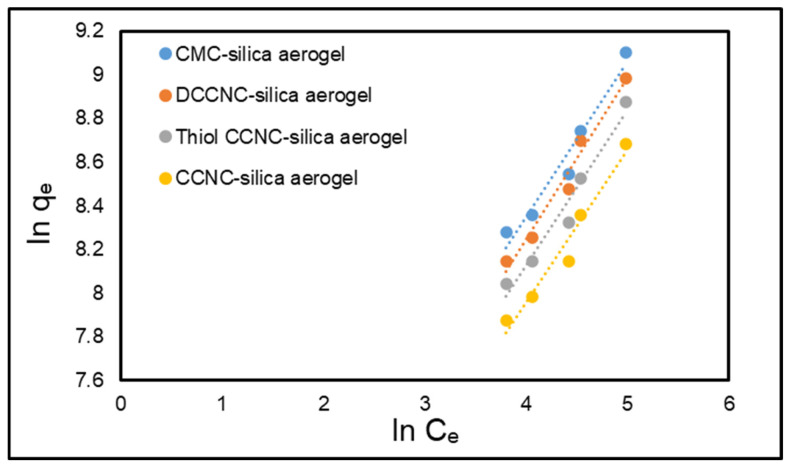
Freundlich isotherm model for the adsorption of MB onto functionalized cellulose-based silica aerogels.

The Freundlich constants K_*F*_ (adsorption capacity) [(mg/g) ((Lmg^−1^)^1/*n*^)] and *n* (adsorption intensity) can be derived from the intercept and slope of the ln *qe* versus ln *Ce* plot. Figure 12 and Figure 13 present the fitting plots for the Langmuir and the Freundlich adsorption isotherms, respectively. Table 5 presents a summary of the parameters that can be ascertained through these isotherm models. The Langmuir isotherm exhibited a significantly higher correlation coefficient (R^2^) compared to the Freundlich isotherm (Table 5). The adsorption of MB dye onto functionalized cellulose-based silica aerogels is more consistent with the Langmuir isotherm model. The theoretical *q*_*m*_ derived from the Langmuir model closely approximated the experimentally determined value.

**Figure 14:**
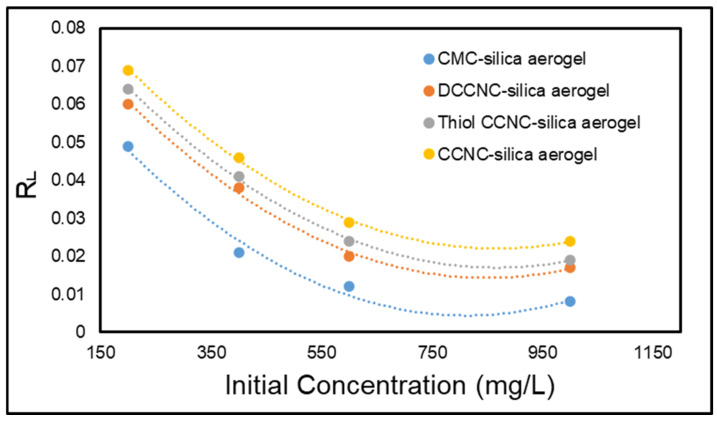
Separation factor for the adsorption of MB onto functionalized cellulose-based silica aerogels.

Furthermore, the *R*_*L*_ values determined via Equation (6) were plotted in relation to the initial concentration of MB dye, as illustrated in Figure 14. The *R*_*L*_ values varied between 0.0081 and 0.069, indicating a favourable interaction between the adsorbent and MB dye molecules. The *R*_*L*_ values approaching zero suggest that the adsorption of MB dye onto functionalized cellulose-based silica aerogels is advantageous at both low and high initial MB concentrations.

### 3.8. Thermodynamic Studies

The impact of temperature variation on the adsorption of MB dye on functionalized cellulose-based silica aerogels was evaluated, and the nature of this process was investigated. Fundamental thermodynamic parameters (ΔH°, ΔS°, ΔG°) were estimated using the following equations [65].

(8) $$lnKc=\left(\frac{∆S{}^{\circ}}{R}\right)-\left(\frac{∆H{}^{\circ}}{RT}\right)$$

(9) ∆G°=−RT lnKc

**Figure 15:**
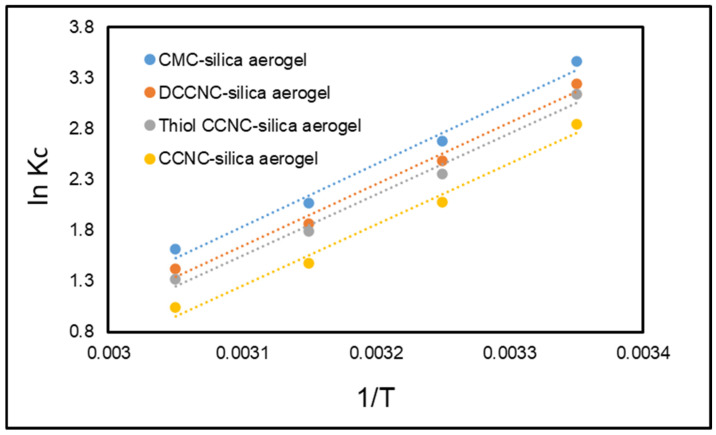
Van’t Hoff plot for the adsorption of MB onto functionalized cellulose-based silica aerogels.

Kc denotes the equilibrium constant (dimensionless), while ΔH°, ΔG°, and ΔS° signify the standard enthalpy change, standard Gibbs free energy change, and standard entropy change, respectively. The universal gas constant, denoted as R, is 8.314 × 10^−3^ KJ·mol^−1^·K^−1^, while T represents the temperature measured in Kelvin. The values of ΔH° and ΔS° were obtained from the intercept and slope of the ln Kc versus 1/T plot (Figure 15). Table 6 presents the thermodynamic parameters for the adsorption of MB dye onto functionalized cellulose-based silica aerogels.

The process is exothermic because the Kc values decrease as the temperature increases, and the ΔH° value is negative. The extent of ΔH° depends on the kinds of forces that are at work during adsorption. ΔH° values between 2 and 40 kJ/mol show van der Waals forces, dipole bonding, hydrogen bonding, and/or coordination exchange. ΔH° values above 60 kJ/mol, on the other hand, show that a chemical bond is forming. The ΔH° value found in this study (−16.20 to −39.22 kJ/mol) shows that the physical adsorption process is exothermic. Also, the change in entropy (ΔS°) was negative (−54.39 to −107.40 J/mol·K), which means that the process is more likely to happen at lower temperatures. Also, the fact that the negative values of ΔG° get smaller as the temperature rises shows that the process happens more naturally at lower temperatures.

### 3.9. Kinetic Studies

To enhance the understanding of the adsorption process of MB dye onto CCNC silica composite aerogel, the widely used pseudo-first-order, pseudo-second-order, and intra-particle diffusion kinetic models were employed and are outlined as follows [66,67]:

(10) $$\ln\left(q_{e}-q_{t}\right)=\ln q_{e}-k_{1}*t$$

(11) $$\frac{t}{q_{t}}=\frac{1}{K_{2}}+\frac{t}{q_{e}}$$

- *q*_*e*_ is the adsorption amount at equilibrium;
- *q*_*t*_ (mg/g) is the amount of MB dye adsorbed at time *t*;
- *k*_1_ (min^−1^) is the first-order rate constant;
- *k*_2_ (g/mg·min) is the second-order rate constant;
- *k*_*id*_ is the intra-particle diffusion rate constant (mol/g·min^1/2^);
- *C* indicates the boundary-layer thickness.

**Figure 16:**
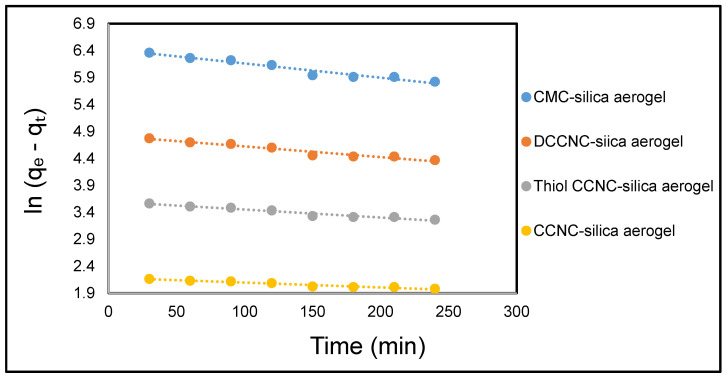
Kinetic models showing pseudo-first order for the adsorption of MB onto functionalized cellulose-based silica composite aerogel.

**Figure 17:**
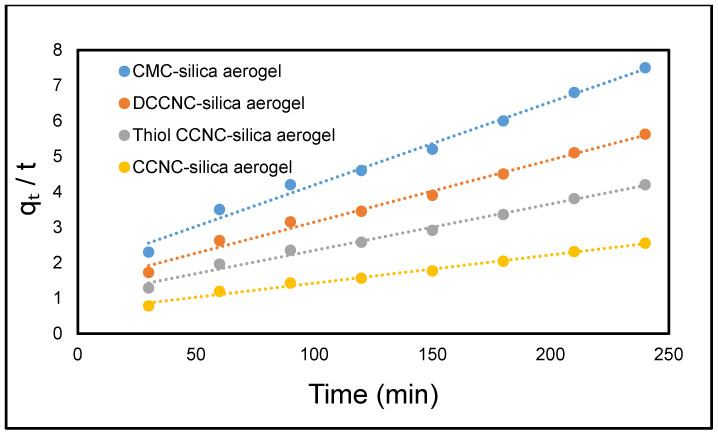
Kinetic models showing pseudo-second order for the adsorption of MB onto functionalized cellulose-based silica composite aerogel.

Figure 16 and Figure 17 illustrate the linear representations of the pseudo-first-order and pseudo-second-order models, respectively. The parameters derived from these linear plots are presented in Table 7. Upon comparison of the determination coefficients R^2^, the pseudo-first order model (R^2^ = 0.9230–0.9507) was found to be less effective than the pseudo-second-order model (R^2^ = 0.992–0.995). The calculated equilibrium capacity values (*qe*, *cal*) derived from the pseudo-second-order model were more closely aligned with the experimental values (*q*_*e*_, *exp*) compared to those obtained from the pseudo-first-order model. This suggests that the adsorption of MB dye onto functionalized cellulose-based silica aerogels is more accurately represented by the pseudo-second-order model.

### 3.10. Cellulose-Based Silica Composite Aerogel Reusability

**Figure 18:**
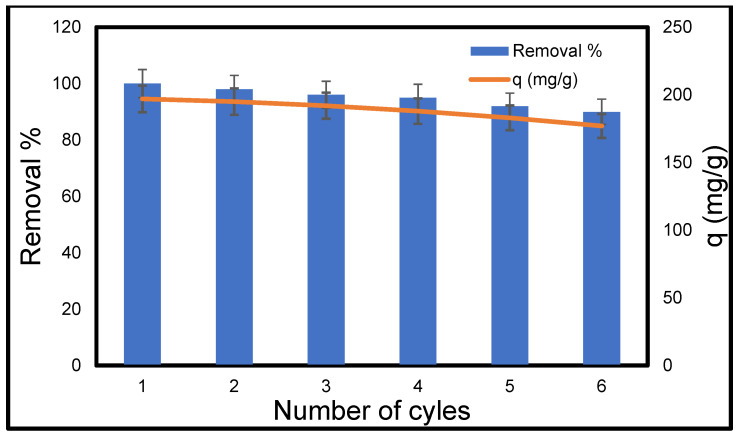
Reusability of CMC silica composite aerogel for the adsorption of MB. [Conc. 200 mg/L, Volume 50 mL, pH 10, Mass 0.05 g, and Temperature 25 °C].

The economy needs to have an adsorbent that is stable and easy to replace. So, it is very important that an adsorbent can be recycled. Based on this idea, CMC silica composite aerogel was put through several cycles of adsorption and desorption (other materials were not subjected to reusability studies). Figure 18 shows that the removal efficiency stayed above 90%, and after six cycles in a row, the adsorption capacity reached a maximum of 177 mg/g. This shows that the made CMC silica composite aerogel is very stable and can be used repeatedly.

### 3.11. Performance Comparison with Other Adsorbents

This study assesses the efficacy of functionalized cellulose–silica aerogels by comparing their methylene blue (MB) adsorption capacity with that of other recently investigated adsorbents, as detailed in Table 8.

The CMC-silica aerogel exhibits a capacity of 197 mg/g, indicating its effective performance. It surpasses various other materials derived from cellulose or composites. For example, it can hold considerably more than chitosan cross-linked graphene oxide/calcium alginate composites (181.81 mg/g) [56] and magnetic cellulose/Fe_3_O_4_ beads (80.19 mg/g) [54]. Fan et al. (285.2 mg/g) [24] presented a functionalized cellulose-based aerogel and various graphene oxide composites demonstrating superior capabilities. However, the primary advantages of the CMC-silica aerogel developed in this work extend beyond its high adsorption capacity.

Sustainability and Cost-Effectiveness: The aerogel is synthesised from sugarcane bagasse, an abundant and low-cost agricultural waste, promoting resource recovery and a circular economy. In contrast, adsorbents relying on graphene oxide or complex cross-linking agents often involve more expensive, energy-intensive, or less environmentally benign precursors and synthesis routes.

Superior Regenerative Performance: A key practical advantage is the material’s exceptional reusability. As demonstrated in Section 3.10, the CMC-silica aerogel retained over 90% of its original efficiency after six consecutive adsorption-desorption cycles. This robust regenerability reduces operational costs and solid waste generation, a critical factor for industrial application that is not commonly reported or achieved to this extent for many alternative adsorbents.

Synergistic Material Properties: The performance stems from a synergistic combination of a favourable mesoporous structure (pore diameter 14.33 nm) for dye diffusion and a high density of accessible carboxylate groups for strong electrostatic interactions. While some compared materials may excel in one aspect (e.g., surface area), the CMC-silica aerogel offers a balanced and effective combination of properties tailored for cationic dye removal.

## 4. Conclusions

This research presents the synthesis and utilization of functionalized cellulose-based silica aerogels for the efficient adsorption of methylene blue (MB) dye from aqueous solutions. Carboxylate (CCNC), double carboxylate (DCCNC), carboxymethyl (CMC), and thiol-functionalized cellulose nanocrystals were incorporated into silica aerogel matrices. This resulted in nanocomposites demonstrating enhanced structural and adsorptive properties. The CMC-silica aerogel exhibited the maximum adsorption capacity of 197 mg/g and achieved a removal efficiency of 100% under ideal conditions, namely at pH 10, 25 °C, and a contact duration of 60 min. The observed performance results from the elevated quantity of carboxyl groups, which improve electrostatic interactions with cationic MB molecules. The BET analysis demonstrates a favourable mesoporous structure.

The adsorption process conformed to the Langmuir isotherm and pseudo-second-order kinetics, indicating monolayer adsorption and chemisorption as the rate-limiting step. Thermodynamic analyses indicated that adsorption occurs in an exothermic and spontaneous manner. The CMC-silica aerogel exhibited notable reusability, sustaining a removal efficiency exceeding 90% after six cycles of adsorption and desorption. The results indicate that functionalized cellulose-based silica aerogels may serve as effective, durable, and reusable adsorbents for the removal of cationic dyes from industrial effluents. This would assist in environmental remediation and water purification technology.

This study possesses many limitations, despite the favourable outcomes. The research was conducted in a regulated laboratory setting using a single model pollutant (MB) in distilled water. The efficacy and stability of the adsorbents in complex industrial effluent, characterized by various ions, organic matter, and fluctuating pH levels, remain uncertain. Furthermore, the synthesis process must be scalable, and a comprehensive economic analysis is required for practical use.

Therefore, future research must focus on evaluating adsorption efficiency in genuine industrial effluent samples, investigating selectivity and competitive adsorption among common water pollutants (e.g., various dyes or heavy metals), and assessing long-term stability and performance in continuous-flow column experiments. It is essential to examine the life-cycle assessment and production costs on a broader scale to prepare the technology for commercial application.

## Figures and Tables

**Table 1:** Crystalline index values of materials analyzed through Segal and deconvolution methods.

Fiber Type	CI (%) (Segal)	CI (%) (Deconvolution)
Raw SCB	29	27
Extracted cellulose	66	63
CCNC	58	54
DCCNC	46	44
CMC	24	21

**Table 2:** The crystalline index values of cellulose (PFA) nanocomposites were analyzed using deconvolution methods.

Fiber Type	CI (%) (Deconvolution)
CCNC silica aerogel	42
DCCNC silica aerogel	34
Thiol CCNC silica aerogel	29
CMC silica aerogel	26

**Table 3:** Physical parameters of the prepared coagulants.

Sample	Specific Surface Area (m^2^/g)	Pore Volume (cm^3^/g)	Pore Diameter (nm)
CMC-SiO_2_	195.06	0.70	14.33
DCCNC-SiO_2_	446.86	0.53	4.59
CCNC-SiO_2_	312.00	0.60	7.70
Thiol CCNC silica	280.12	0.55	7.90

**Table 4:** Adsorption study results for all functionalized cellulose-based silica aerogels.

Cellulose-Based Silica Aerogels	Removal (%)	Adsorption Capacity (mg/g)
CCNC silica	63	32
DCCNC silica	74	37
Thiol-CCNC silica	74	37
CMC-silica	100	197

**Table 5:** Adsorption isotherm parameters for the adsorption of MB onto the studied functionalized cellulose-based silica composite aerogel.

	Langmuir Model			Freundlich Model		
Material	q_max_ (mg/g)	K_L_ (L/mg)	R^2^	K_F_ (mg/g)	*n*	R^2^
CCNC silica	31.95	0.15	0.990	36.48	3.33	0.904
DCCNC silica	36.10	0.19	0.990	41.42	3.45	0.908
Thiol CCNC silica	36.05	0.19	0.995	40.51	3.42	0.896
CMC silica	197.33	0.33	0.994	61.45	3.61	0.892

**Table 6:** Thermodynamic parameters for the adsorption of MB onto the studied functionalized cellulose-based silica composite aerogel.

Material	T(K)	ΔG° (KJ·mol^−1^)	ΔH° (KJ·mol^−1^)	ΔS° (J·mol^−1^·K^−1^)
CCNC silica	298	−25.12	−16.20	−54.39
DCCNC silica	298	−19.42	−16.84	−58.89
Thiol CCNC silica	298	−20.16	−16.60	−57.42
CMC silica	298	−8.55	−39.22	−107.40

**Table 7:** Kinetic parameters for the adsorption of MB onto the studied functionalized cellulose-based silica composite aerogel.

Material	C_o_ (mg/L)	*q*_*e*,exp_ (mg/g)	Pseudo-First Order			Pseudo-Second Order		
			*q*_*e*,cal_ (mg/g)	*k*_1_ (min^−1^)	R^2^	*q*_*e*,cal_ (mg/g)	*k*_2_ (g/mg·min)	R^2^
CCNC silica	200	32	24	0.0026	0.9507	28	0.000989	0.993
DCCNC silica	200	37	25	0.0024	0.9494	34	0.000741	0.993
Thiol CCNC silica	200	37	25	0.0024	0.9489	34	0.000723	0.992
CMC silica	200	197	182	0.0174	0.9652	199	0.000242	0.995

**Table 8:** Comparison of the maximum MB adsorption capacity of CMC-silica aerogel with other adsorbents reported in the literature.

Adsorbent Material	Maximum Adsorption Capacity (mg/g)	Reference
CMC-Silica Aerogel (This work)	197	This work
CCNC-silica Aerogel (This work)	32	This work
DCCNC-silica Aerogel (This work)	37	This work
Thiol-CCNC-silica Aerogel (This work)	37	This work
Functionalized Cellulose-based Aerogel	285.2	[24]
Chitosan–graphene oxide/calcium alginate	181.8	[56]
Magnetic Cellulose/Fe_3_O_4_ Beads	80.19	[54]
Regenerated Cellulose/Graphene Oxide Aerogel	176.5	[53]
Carboxymethyl Cellulose/Carboxylated Graphene Oxide	192.7	[46]
Chitosan-crosslinked-poly(alginic acid) nanohydrogel	94.3	[59]

## Data Availability

The raw data supporting the conclusions of this article will be made available by the authors on request.
